# Simple method for fabricating scattering layer using random nanoscale rods for improving optical properties of organic light-emitting diodes

**DOI:** 10.1038/s41598-018-32538-4

**Published:** 2018-09-25

**Authors:** Jin Ho Kwack, Junhee Choi, Cheol Hwee Park, Ha Hwang, Young Wook Park, Byeong-Kwon Ju

**Affiliations:** 1 Display and Nanosystem Laboratory, School of Electrical Engineering, Korea University Seoul, Seoul, 136-713 Republic of Korea; 2 Samsung Display Co., Samsung St. 181, Tangjeong-Myeon, Asan-City, Chungcheongnam-do 31454 Republic of Korea; 3 School of Mechanical and ICT Convergence Engineering, SUN MOON University, Asan-City, Chungcheongnam-do 31460 Republic of Korea

## Abstract

We investigated a low-temperature mask-free process for preparing random nanoscale rods (RNRs) as a scattering layer. The process involves spin coating and dry etching, which are already widely applied in industry. Our film exhibited 17–33% optical haze at 520 nm wavelength and 95% total transmittance in the visible range. Therefore, this film can be used as a scattering layer for improving viewing angle characteristics and decreasing substrate mode loss in organic light-emitting diodes (OLEDs). Specifically, we focussed on varying the height and density of the RNRs to control the optical characteristics. As a result, the OLEDs with RNRs revealed a variation in colour coordinates of Δ(x, y) = (0.007, 0.014) for a change in the viewing angle, which was superior to those without the RNRs that displayed a variation of Δ(x, y) = (0.020, 0.034) in CIE 1931. Moreover, the OLEDs with RNRs exhibited 31% enhanced external quantum efficiency compared to those of the OLEDs with the bare substrate. The flexibility of the polymer used for the RNRs and the plasma treatment suggests that the RNRs can be applied to flexible OLED displays and lighting systems.

## Introduction

Organic light-emitting diodes (OLEDs) are being widely applied in displays for mobiles and televisions owing to their self-emitting characteristics, excellent colour gamut, high-speed operation, and applicability in flexible or stretchable devices^1^. Practical OLEDs function based on phosphorescence owing to their nearly 100% internal phosphorescence efficiency^2,3^. However, the external quantum efficiency of OLEDs is ~20% because the surface plasmon polariton modes at the metal-organic interface, the waveguide mode in the indium tin oxide (ITO)/organic layer, and the substrate mode in the glass substrate produce light loss^4–8^. Generally, light loss in the substrate mode is larger than that in the waveguide mode due to small differences in refractive index between the ITO/glass interface and the glass/air interface^9^. In addition, microcavity top-emitting organic light-emitting diodes (TEOLED) without the loss of the substrate mode and having the advantage of improved colour purity and aperture ratio have been applied to mobile displays. However, microcavity TEOLED originally had a problem associated with viewing angles due to the blue shift that occurred with the variation of the sight angle, even though the light efficiency along the normal direction was amplified. Therefore, researchers have attempted to find solutions for improving the light extraction and viewing angle of OLEDs^10^.

Different approaches have been adopted with the aim of optimizing light extraction and viewing angle, including micro lens array (MLA)^5,11–16^, dielectric Bragg gratings^17–19^, surface plasmons^20^, buckling patterns^21^, periodic corrugation^8^, low-index grids^22^, and photonic crystals^23^. Methods that modify the internal or external surfaces of the substrate of OLEDs minimize the total internal reflection. Such surface shapes are produced by different technologies which traditionally involve photo-lithography or printing, moulding, and embossing methods^24–28^. Therefore, these technologies employ at least the photo-lithographic step or utilize lithographic templates once. They generally end up being considerably complex or expensive for mass production. On the other hand, other researchers have reported a simple scattering layer synthesized by competitive and scalable methods. In these methods, particles or voids having different refractive indices were embedded in a polymer matrix and utilized for the scattering layer^29–36^. However, because the particles or voids are micro-sized or the pitch between the nearest particles or voids is micro-sized, these techniques reveal a small effect of diffraction that is difficult to control. In addition, to prevent the loss of the substrate mode, the most representative MLA has larger visibility than a nano-sized array because of its size. Alignment is a problem in the case of high-resolution displays with very small pixels.

In this paper, we propose a simple method to fabricate random nanoscale rods (RNRs) as scattering layers for enhancement of light extraction and improvement of viewing angle characteristics in OLEDs. In our device, there is virtually no spectral distortion or shift, which are issues observed in 2D photonic crystal-based OLEDs, and the spectral shift according to the change in viewing angle is reduced. This layer is fabricated by a cost-effective and scalable procedure that consists of coating and etching the polymer at low temperatures (below 100 °C) without photomasks or templates. Additionally, the RNRs can control the diffraction as well as scattering effects in the visible wavelength range owing to the distance between closest rods and are compatible with high-resolution displays for virtual reality because the process of alignment between the individual pixels in the panel and the scattering layer is unnecessary. The RNRs can also be applied in flexible displays and lighting owing to the use of etched polymers. OLEDs equipped with RNRs exhibit superior optical properties such as high external quantum efficiency (EQE), high luminance efficiency (LE), and colour variation with changes in viewing angle, to control OLEDs.

## Results and Discussion

**Figure 1:**
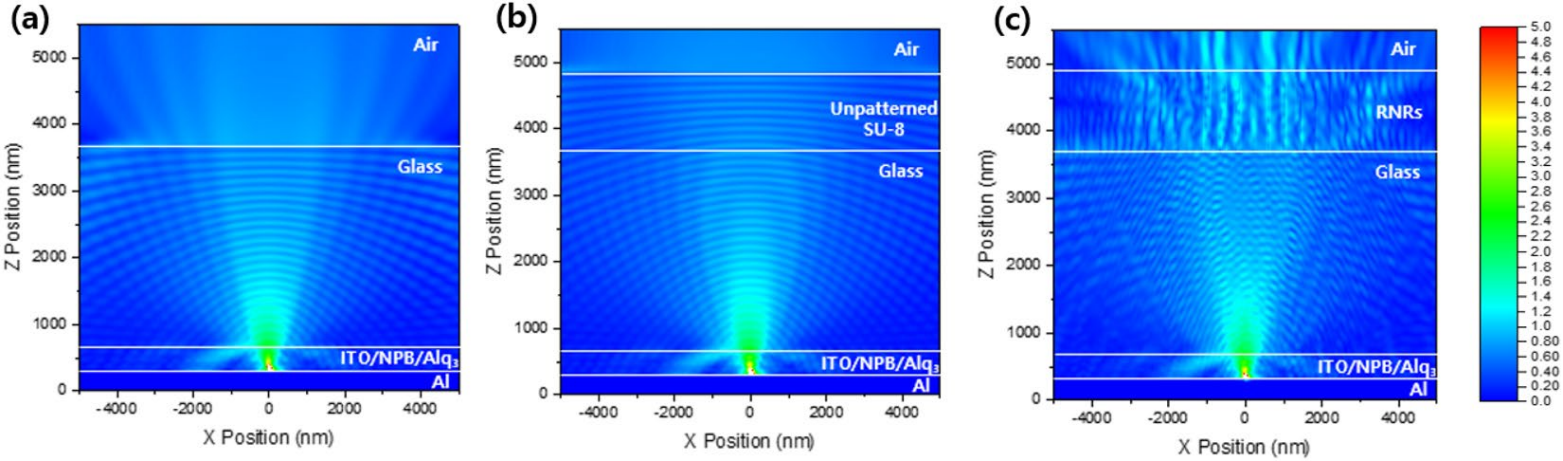
FDTD simulation of E-field distribution induced by transverse magnetic and electric dipoles: (**a**) Reference (w/o RNRs), (**b**) unpatterned SU-8 on a glass structure, and (**c**) RNRs on the glass structure.

**Figure 2:**
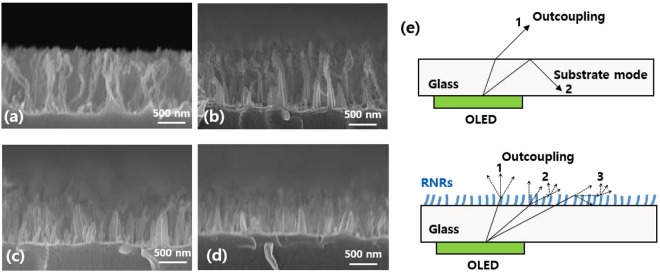
Cross-sectional scanning electron microscopy images of random nanoscale rods (RNRs) obtained under different plasma etching conditions: (**a**) RNRs 1, (**b**) RNRs 2, (**c**) RNRs 3, and (**d**) RNRs 4, and (**e**) schematic illustration of OLED outcoupling enhancement by the RNR scattering layer.

To investigate the optical effect of RNRs on glass, the electrical field distribution was simulated by the finite-difference time domain (FDTD) method. The boundary conditions for the simulation were set for a perfect optically matched layer to avoid the reflection of electromagnetic waves at the edges of the structure on all the sides, except for the metal cathode layer. The simulated structure consisted of an aluminium cathode, *N*,*N*’-bis(naphthalen-1-yl)-*N*,*N*’-bis(phenyl)-benzidine (NPB; refractive index n = 1.81), tris(8-hydroxy-quinolinato)aluminium (Alq_**3**_; n = 1.72), ITO (n = 1.9), glass (n = 1.52), SU-8 polymer, and RNRs (n = 1.59). A dipole source of visible wavelength was generated in the Alq_**3**_ layer, and one transverse electric (TE) and two transverse magnetic (TM) modes were used^37^. To reflect the randomness of RNRs in the simulation, their height was varied from 1000 nm to 1200 nm and width from 50 nm to 150 nm (Supplementary Information Fig. S1). In the reference structure, most of the light incident at an angle above the critical angle became a waveguide because of the difference in refractive index between glass and air (Fig. 1a). In order to confirm the optical effect of the SU-8 polymer, a simulation using SU-8 without the pattern was performed (Fig. 1b). As a result, the optical path was almost unchanged, and the difference between the refractive indices of glass and air slightly increased; this difference seemed to be slightly adversely affected for external light extraction. In contrast, in the RNRs present on the glass structure, some portions of the waveguide light were allowed to escape from the glass substrate, and light extraction increased below the critical angle (Fig. 1c). Optical phenomena such as refraction, diffraction, and interference were produced by the RNRs because the differences in refractive index at the interface between glass and air changed and the random periodic rods revealed distances between each other that were similar to the visible wavelength. Figure 2e illustrates the light path in OLEDs. The photons trapped (rays 2 and 3) within the glass are scattered by the RNRs, increasing the probability of them being emitted from the structure. Furthermore, as a result of simulation, in which scattering occurs several times in one rod, it can be inferred that scattering occurs more frequently when the height of the rod is greater.

It is desirable to fabricate a nanostructure of proper height and periodicity that is comparable to the wavelength of the visible range in order to enhance light extraction. For manufacturing RNRs, SU-8 polymer was chosen because it is highly transparent to visible light and has a higher refractive index than glass. Figure 2 shows the cross-sectional scanning electron microscopy images of the RNRs obtained under different plasma etching conditions. Obtained by using only an oxygen plasma for a duration of 9 min (denoted as RNRs 1), the structure was random columnar-like with high aspect ratio and density^38^. The height and density of RNRs 1 were about 1200 nm and 9.7 ea/µm^2^, respectively (Fig. 2a, Supplementary Information Fig. S2a). The width of the individual rods was observed to be approximately 50 nm. To control the height and density, we applied an additional argon plasma treatment at higher powers and lower process pressures than the oxygen plasma. The low pressure led to an increase in the mean free path of argon ions and reduced the energy loss of these ions as they approached the RNRs. In general, the etching process using argon plasma carried out in a reactive ion etcher resulted in anisotropic and directional features due to the utilization of the physical bombardment energy^38^. As a result of these features, the height and density were adjusted as much as a target to the little changed width of the RNRs. The three treated RNRs shown in Fig. 2b–d corresponded to argon plasma treatment durations of 3, 6, and 9 min (denoted as RNRs 2, RNRs 3, and RNRs 4, respectively), while the conditions of the oxygen plasma process remained unchanged. As the duration of the argon plasma treatment increased, the height decreased to 950, 580, and 440 nm, respectively, and the density of the RNRs reduced (Supplementary Information Fig. S2).

**Figure 3:**
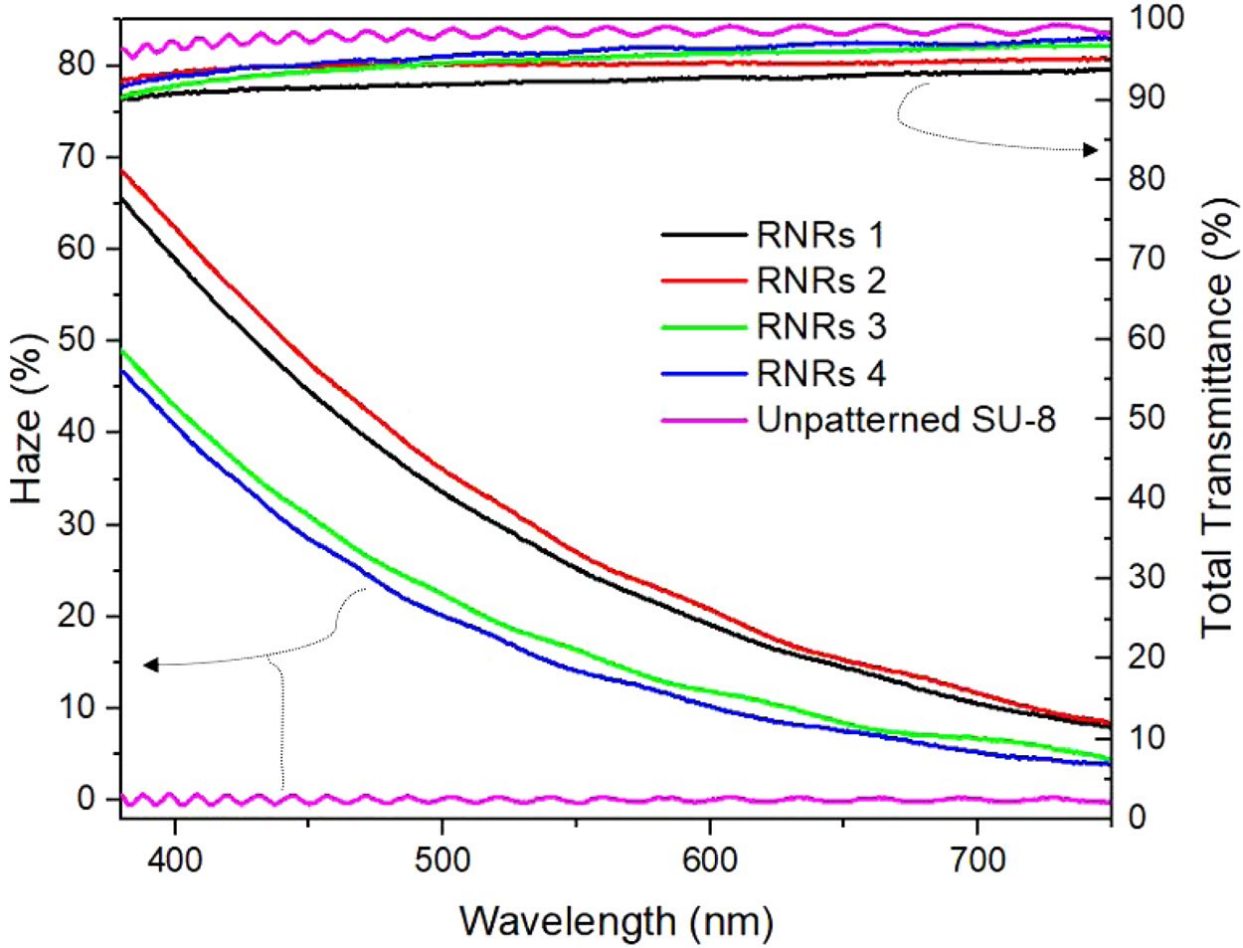
Total transmittance and haze as functions of visible wavelength of different RNRs and unpatterned SU-8.

Figure 3 shows the total transmittance and haze as functions of wavelength for the different RNRs. According to the material data sheet provided by Micro-Chem., SU-8 is highly transparent with little absorption in the visible range. The optical properties of unpatterned SU-8 fabricated by spin coating showed that the total transmittance was over 95% while the haze was less than 1%. All the RNRs exhibited total transmittance values above 90%. Therefore, the RNRs were thought to be appropriate films for light extraction, with minimal light loss through absorption and back scattering. The haze slightly increased from 30.1% (RNRs 1) to 31.5% (RNRs 2) at 520 nm for an additional argon plasma time of 3 min and decreased to 19.4% (RNRs 3) and 17.8% (RNRs 4) for treatment times of 6 min and 9 min. Haze was calculated using equation (1).

1 $$Haze=\frac{Total\,transmittance-specular\,transmittance}{Total\,transmittance}$$

In the case of RNRs 1, haze was affected by the reduced total transmittance, which was attributed to increases in back scattering and the amount of trapped light in RNRs when the height of the rod was greater than a specific thickness. Besides, as the shapes of rods were longer, it was not perfectly straight, and therefore, back-scattering and light trapping seemed to occur more than in the cases of the other RNRs. The density of the rods also had an effect on the total transmittance, and RNRs 1, with the highest density, seemed to be one of the reasons for the decrease in the total transmittance. In the cases of RNRs 3 and RNRs 4, the lower heights and densities led to decreases in haze due to lower scattering probabilities. Considering the total transmittance and haze, the treatment condition of RNRs 2 was expected to produce the optimal height and density of rods for enhanced light extraction of OLEDs.

**Figure 4:**
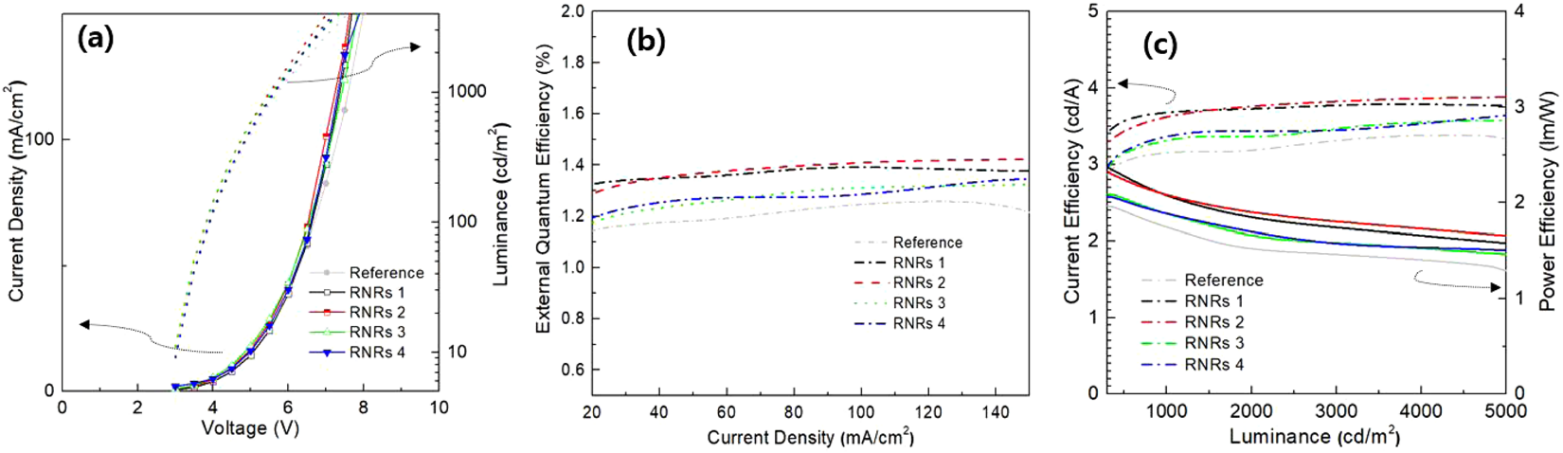
EL characteristics of OLEDs with different RNRs: (**a**) J-V and L-V characteristics, (**b**) external quantum efficiencies as functions of current density, and (**c**) current and power efficiencies as functions of luminance.

The electrical characteristics of the device with and without the RNRs were almost identical since they were placed, where they do not affect the devices (Fig. 4a). The OLED with unpatterned SU-8 showed the identical electrical properties and a slightly lower external quantum efficiency (EQE; Supplementary Information Fig. S3). The lower EQE is attributed to the fact that the refractive index of the SU-8 film is higher than that of the glass and that weak interfacial reflections occur between the glass and the SU-8 film. However, the OLEDs with the RNRs exhibited higher efficiencies than those without the RNRs in terms of overall current density. The OLED treated with oxygen plasma for 9 min and argon plasma for 3 min (RNRs 2) showed the maximum EQE of 1.42%, while the reference device exhibited an EQE of 1.26%. The enhancement in the maximum EQE was 13% in the normal direction. Considering the scattering characteristics, the films with RNRs 1 and RNRs 2 were similar, but it was estimated that the difference in total transmittance caused a gap in the EQE. The maximum EQEs of RNRs 1, 3, and 4 were 1.4%, 1.32%, and 1.34%, respectively, which increased relative to the reference device. The current and power efficiencies of RNRs 2 were improved by 3.89 cd/A and 1.63 lm/W, respectively, relative to the values of 3.39 cd/A and 1.42 lm/W of the reference device. RNRs 1, 3, and 4 showed values of 3.81 cd/A, 3.58 cd/A, 3.64 cd/A and 1.71 lm/W, 1.50 lm/W, 1.52 lm/W, respectively.

Table 1 shows the densities and average pitches of the RNRs calculated using image processing software (Image-Pro Plus 4.5, Media Cybernetics, Inc.). The densities of RNRs 1, 2, 3, and 4 were 9.7 ea/μm^2^, 7.1 ea/μm^2^, 5.1 ea/μm^2^, and 3.5 ea/μm^2^, respectively, and decreased with the increase in argon plasma time. The reduction in density from RNRs 1 to RNRs 2 was 2.6 ea/μm^2^, whereas that from RNRs 3 to RNRs 4 was 1.6 ea/μm^2^. It was presumed that this was due to the initial removal of RNRs that were not straight-shaped among the RNRs etched by the oxygen plasma. The average pitch was derived from the density and calculated using the distance between the centre of a certain rod and the centre of the nearest rod, assuming that the rod was circular in shape. Though the distances between the RNRs were not always identical due to random periods, the calculated average distance between closest rods suggested that RNRs 2 more likely produced diffraction than the other rods^21^.

**Table 1:** Density, calculated average pitch, total transmittance, haze, and the enhancement in EQE for different RNRs.

	RNRs 1	RNRs 2	RNRs 3	RNRs 4
Density (ea/μm^2^)	9.7	7.1	5.1	3.5
Calculated Average Pitch (nm)	361	423	500	603
Total Transmittance (%)	92.2	94.5	94.8	95.8
Haze (%)	30.1	31.5	19.4	17.8
Enhancement in EQE (%)	21.0	23.4	9.2	12.5

**Figure 5:**
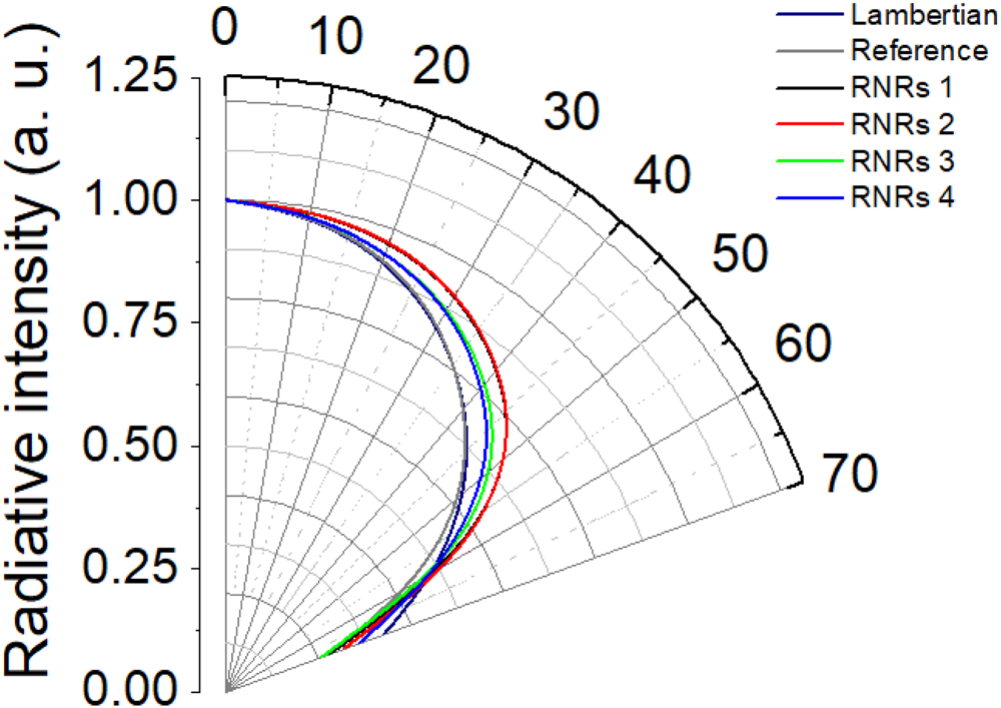
Normalized angular luminance distributions of OLEDs operating at 7 mA between 0° and 70°.

Figure 5 shows that the OLEDs with RNRs exhibited improved luminance intensities with changes in viewing angle from 0° to 70° due to scattering effect. The luminance was measured with a constant current of 7 mA, and the reference device had a luminance of 578 cd/m^**2**^ in the normal direction. Since the luminance in the normal direction differed according to the RNR condition, it was normalized based on luminance in the normal direction. Under the conditions of RNRs 1 and RNRs 2, the normalized luminance intensity increased by 13.6% and 14.1%, respectively, with respect to the reference device at the angle of 40°, and these two conditions revealed nearly similar viewing angle characteristics from 0° to 70°. The devices with RNRs 3 and RNRs 4 also exhibited enhanced luminances of 9.0% and 7.5%, respectively, compared to the reference device at 40°. It was observed that this viewing angle characteristic showed a similar tendency to the haze values of the RNRs. For the best conditions (RNRs 2), the improvement in the EQE was 23.4% relative to the reference device. The viewing angles of RNRs 1, RNRs 3, and RNRs 4 also increased by 21.0%, 9.2%, and 12.5%, respectively. Therefore, it was confirmed that the efficiency and viewing angle improved by reducing the loss of the substrate mode based on the scattering characteristics of the RNRs. As with the haze properties, the unpatterned SU-8 exhibited almost identical viewing angle characteristics as the reference device (Supplementary Information Fig. S4).

**Figure 6:**
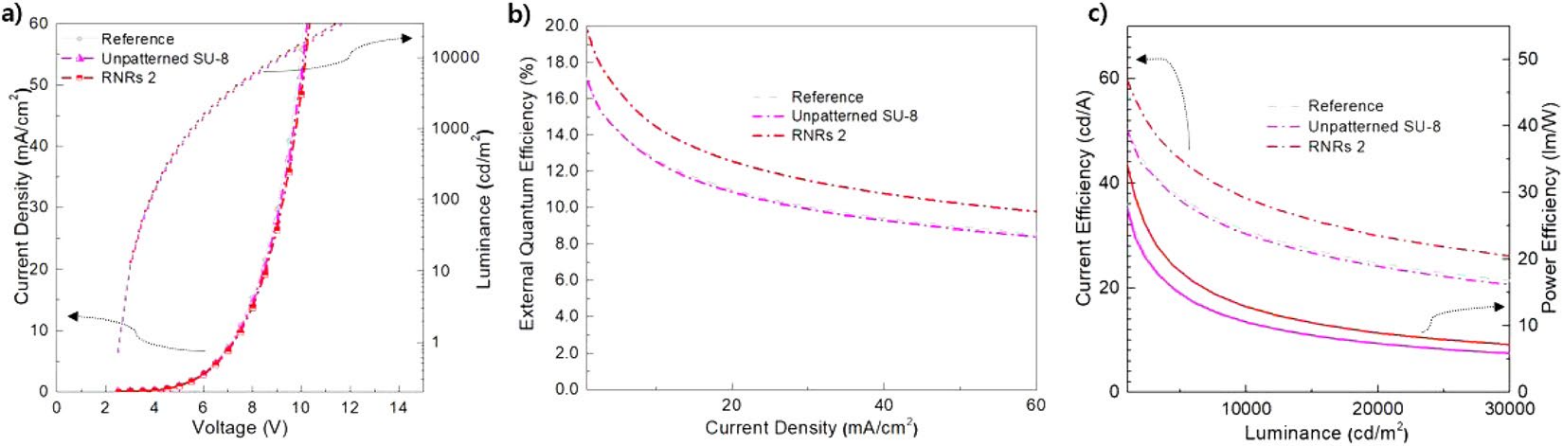
EL characteristics of green phosphorescent OLEDs with RNRs 2, without RNRs (reference), and with unpatterned SU-8: (**a**) J-V and L-V characteristics, (**b**) external quantum efficiencies as functions of current density, and (**c**) current and power efficiencies as functions of luminance.

We also investigated the light extraction efficiency of green phosphorescent OLEDs to re-verify the effect of RNRs due to the low EQE of fluorescent OLEDs with NPB/Alq_3_. The green phosphorescent OLEDs without RNRs, with unpatterned SU-8, and with RNRs 2 (optimal condition) were fabricated. The electrical characteristics of the devices were almost identical (Fig. 6a). However, the OLED with RNRs 2 exhibited higher efficiencies than that without the RNRs or with unpatterned SU-8 in terms of overall current density. The maximum EQE of the OLED with RNRs 2 was 20.92%, while those of the reference and the OLED with unpatterned SU-8 were 17.62% and 17.94%, respectively. The enhancement in the maximum EQE was 18.7% relative to that of the reference device in the normal direction. In addition, the EQEs of the OLEDs with RNRs 2, without RNRs and with unpatterned SU-8 were evaluated as 12.5%, 11%, and 10.8%, respectively, at 20 mA/cm^2^. The enhancement in the EQE was 13.6%, which was similar to the 13% enhancement observed in the case of fluorescent OLEDs. Moreover, the EQE of the OLED with unpatterned SU-8 was similar to that of the reference device, indicating that the SU-8 film did not contribute to the light extraction effect.

**Figure 7:**
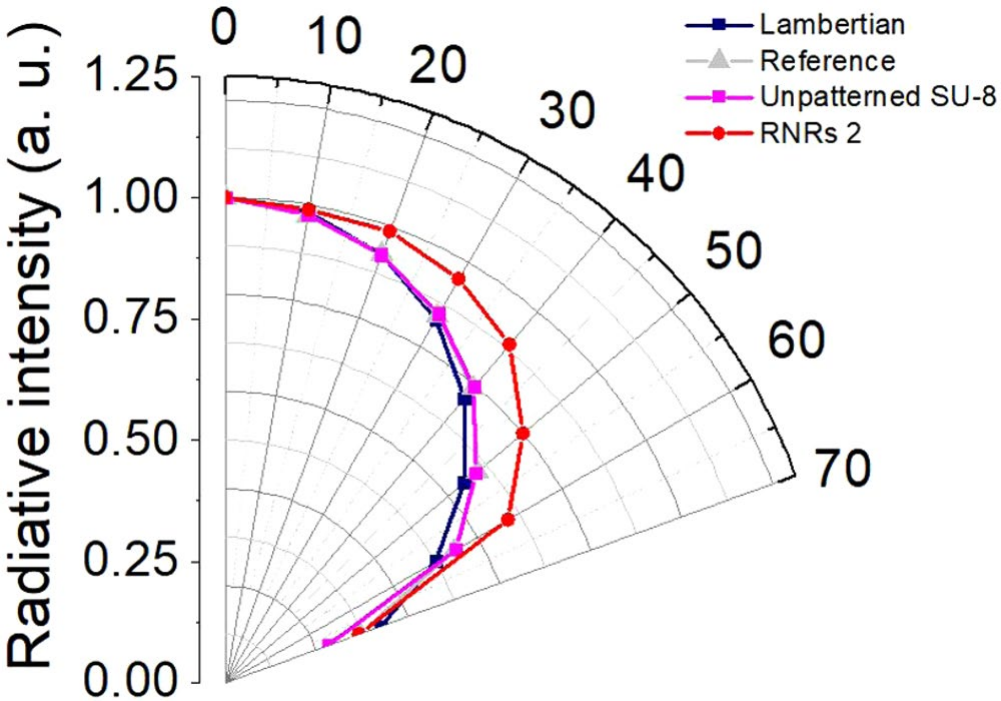
Normalized angular luminance distributions of green phosphorescent OLEDs operating at 0.9 mA.

Figure 7 shows the variations in the luminance intensities of the green phosphorescent OLEDs for three different conditions according to the changes in viewing angle from 0° to 70°. As with fluorescent OLEDs, the different intensities were normalized to the luminance in the normal direction, and the reference device displayed a luminance of 906 cd/m^2^ at the constant current of 0.9 mA. In the case of the green phosphorescent OLED with RNRs 2, the luminance improved relative to that of the reference at all viewing angles due to the scattering effect of RNRs 2. However, in the case of unpatterned SU-8, the change in luminance characteristics according to viewing angle was almost the same as that of the reference. These results show that the scattering effect of the unpatterned SU-8 film is almost non-existent. Considering the viewing angle, the improvement in the EQE of the green phosphorescent OLED with RNRs 2 was 31% relative to that of the reference device. The difference between the EQE improvements of the fluorescence (23.4%) and phosphorescence (31%) OLEDs is presumed to be due to a deviation in the low EQE of the fluorescence device. Furthermore, as a result of the maximum difference in the colour coordinates in CIE 1931 for viewing angles varying from 0° to 70°, the reference device exhibited a variation of Δ(x, y) = (0.020, 0.034), whereas the RNRs 2 device revealed a variation of Δ(x, y) = (0.007, 0.014) (Supplementary Information Fig. S5). This result also proved that the RNRs played appropriate roles as scattering layers.

We demonstrated a simple method for fabricating RNRs as a scattering layer at low temperatures without any mask. In particular, the height and density could be controlled without changing the width of the rods by utilizing the anisotropic etching characteristics of argon plasma. The optimal RNRs showed a haze of 31.5% despite the total transmittance being 94.5%. As a result, a 31% enhancement in the EQE of the OLED was achieved. FDTD simulation and efficiency improvement results indicated that the RNRs were suitable scattering layers that reduced the loss of the substrate mode. Moreover, the variations in light intensity and colour coordinates with viewing angle could be alleviated by using these RNRs. We thus believe that this study will open a new and practical approach to improving the performance of high-resolution displays and flexible lightings.

## Methods

### Numerical simulation

The optical effects of OLEDs with RNRs were simulated using a FDTD software (Lumerical Solutions, Inc.). Each layer had a different refractive index that was measured by a thin-film analyser (F-20, Filmetrics, Inc.). The simulation domain condition involved a perfectly matched layer on all sides, except for the metal cathode layer, where the metal boundary condition was used. The three dipoles (x-, y-, and z-polarized) were placed inside the emitting layer. The optical enhancement image of the OLEDs with RNRs relative to the reference structure was calculated as the ratio of the integrated electric field intensities of the two devices that were measured by a fixed far-field monitor.

### Fabrication

**Figure 8:**
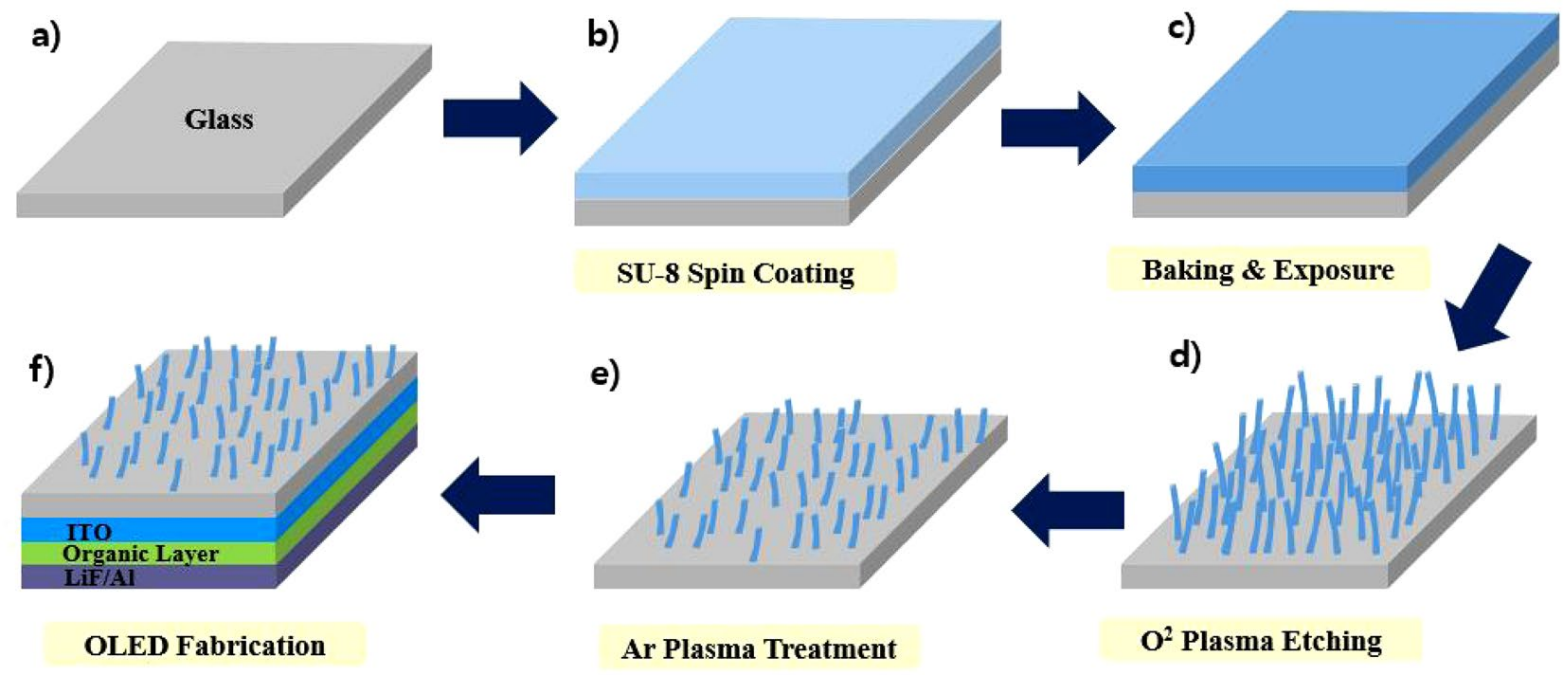
Schematic of the process flow for fabricating OLEDs with RNRs.

The fabrication process of the RNRs as the scattering layer is shown in Fig. 8. The polymer SU-8 (SU-8 2010, Micro-Chem.) was mixed with a thinner in the ratio 1:1 by weight to achieve the target thickness. The mixed material was spin-coated, baked at 95 °C using a hot plate, and exposed at 130 mJ/cm^2^ to conventional UV radiation (350 nm–400 nm). As a result, the thin film was highly transparent to visible light^39^. The film was etched in a reactive ion etcher (RIE) by using oxygen plasma at the RF power of 150 W, process pressure of 70 mtorr, and gas flow of 50 sccm. After the first etching of the polymer, the structures were random columnar-like, with high aspect ratios and densities^40^. A second plasma treatment with argon gas at the high RF power of 200 W and process pressure of 25 mtorr was applied to the structure to reduce the aspect ratio and density. To investigate the properties of the RNRs, organic light-emitting devices were fabricated. To obtain devices with different properties, the duration of argon plasma treatment was altered while the other conditions were unchanged. After the RNRs were formed on glass substrate, ITO was deposited on the opposite side by radio frequency sputtering. The following layers were then thermally evaporated onto the ITO under vacuum conditions (2 × 10^−6^ Torr): a 60 nm thick NPB layer for hole transport, an 80 nm thick Alq_**3**_ layer for light emission, a 0.7 nm thick lithium fluoride layer for electron injection, and a 100 nm thick aluminium layer that acted as the cathode. In the case of green phosphorescent OLEDs, the following layers were utilized: a 40 nm thick NPB(N,N’- bis(naphthalen-1-yl)-N,N’-bis(phenyl)-benzidine) layer and a 10 nm thick TCTA (4,4′,4′′-Tris(carbazol-9-yl)triphenylamine) layer for hole transport, a 30 nm thick CBP(4,4′-bis(carbazol-9-yl)biphenyl) doped with 10 wt% Ir(ppy)3(Tris(2-phenylpyridine)iridium(III)) served as the phosphorescent green emitting layer; and 55-nm-thick B3PyMPM(bis-4,6-(3,5-di-3-pyridylphenyl)-2-methylpyrimidine) as used as the electron-transport layer, a 0.4 nm thick lithium fluoride layer for electron injection, and a 100 nm thick aluminium layer that acted as the cathode.

### Measurements

The surface morphologies and cross-sections of the RNRs were measured by scanning electron microscopy (S-4800, HITACHI High Technology Inc.). The haze and total transmittance were evaluated by UV-vis-NIR spectroscopy (Cary 5000, Agilent Technologies Inc.). The current–voltage characteristics were measured using a Keithley 237 High Voltage Source-Measure Unit (Keithley Instruments, Inc.) and the electroluminescence intensity was measured in a dark box using a spectroradiometer (PR-670 Spectra Scan, Photo Research, Inc.).

## Electronic supplementary material

Supplementary Information
