# Supplementary material for: Simple method for fabricating scattering layer using random nanoscale rods for improving optical properties of organic light-emitting diodes
Source: Sci Rep. 2018 Sep 25;8:14311. doi: 10.1038/s41598-018-32538-4 (PMC6156328; doi:10.1038/s41598-018-32538-4)
Supplement: Supplementary file 1 — Supplementary Information [file 41598_2018_32538_MOESM1_ESM.docx]

**Supplementary Information**

Simple method for fabricating scattering layer using random nanoscale rods for improving optical properties of organic light-emitting diodes

Jin Ho Kwack^1,2^, Junhee Choi^1^, Cheol Hwee Park^1^, Ha Hwang^1^, Young Wook Park^3,*^ and

Byeong-Kwon Ju ^1,*^

^1^Display and Nanosystem Laboratory, College of Engineering, Korea University Seoul 136-713, Republic of Korea

^2^Samsung Display Co., Samsung St. 181, Tangjeong-Myeon, Asan-City, Chungcheongnam-do,31454, Republic of Korea

^3^School of Mechanical and ICT Convergence Engineering, SUN MOON University, Chungcheongnam-do 31460, Republic of Korea

^*^Correspondence and requests for materials should be addressed to Y.W.P. (email: zerook@sunmoon.ac.kr) or B.-K.J. (email: bkju@korea.ac.kr)

Phone No.: +82-2-3290-3665

Fax. No.: +82-2-3290-3791


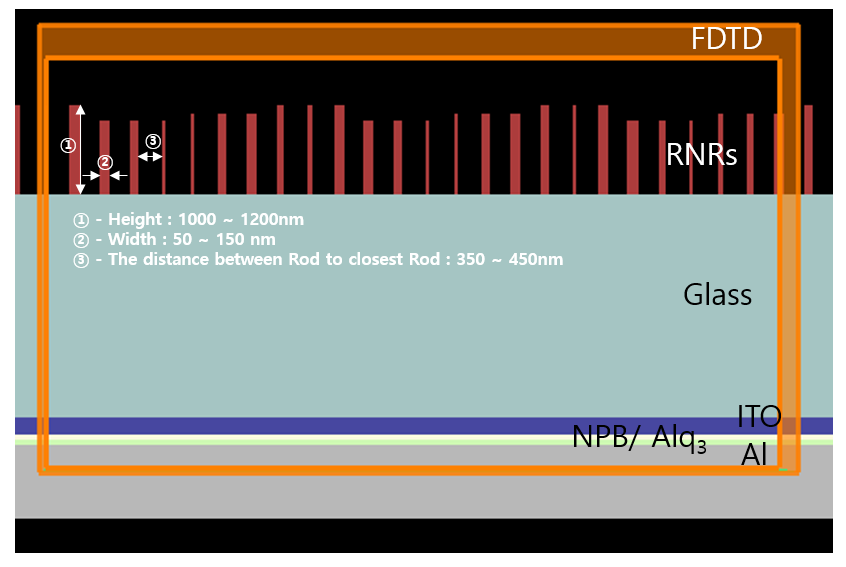


**Supplementary Figure S1**. The structure and detailed RNRs information of FDTD simulation

- The boundary conditions for the simulation were set as a optically perfect matched layer to avoid the reflection of electromagnetic waves at the edges of the structure on all sides, except for the metal cathode layer (Al).

- The heights of RNRs considered were applied 1000, 1100, 1200nm. The widths of RNRs were 50, 100, 150nm. The distance between closest rods was between 350nm and 450nm.

- The FDTD size was x = 10㎛, y = 5.5㎛. Mesh size was applied at x = 8nm, y = 15nm.


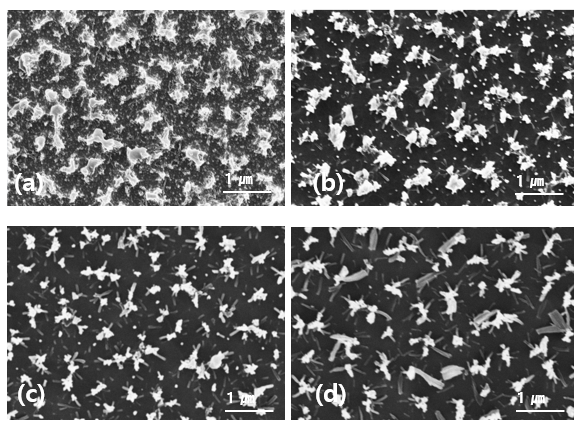


**Supplementary Figure S2**. Scanning electron microscopy plane-view images of random nanoscale rods (RNRs) with different plasma etching conditions: (a) RNRs 1, (b) RNRs 2, (c) RNRs 3, and (d) RNRs 4.


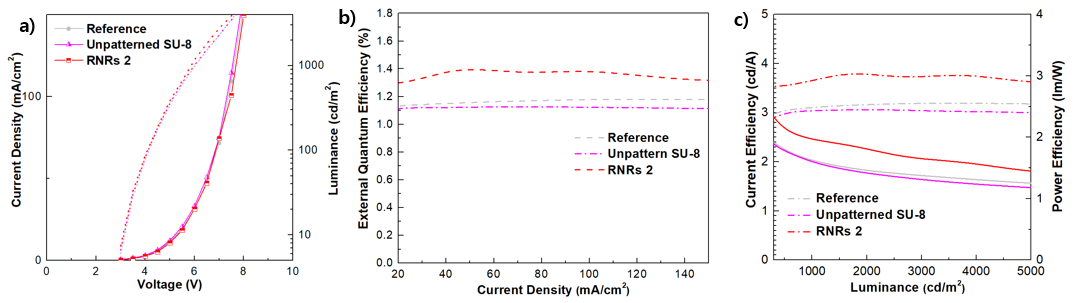


**Supplementary Figure S3**. EL characteristics of the OLEDs with RNRs 2, without RNRs (Reference) and with Unpatterned SU-8: (a) J-V and V-L characteristics, (b) external quantum efficiency as a function of current density, and (c) current and power efficiencies as functions of luminance.


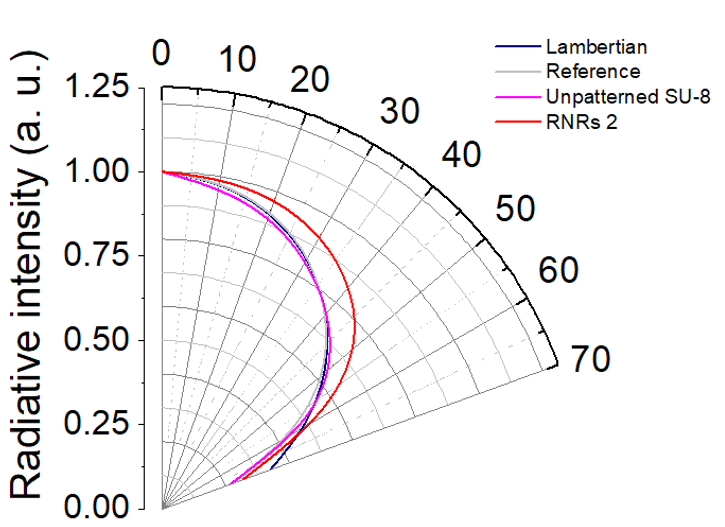


**Supplementary Figure S4**. Normalized angular luminance distribution of OLEDs operating at 7 mA from 0° to 70°.


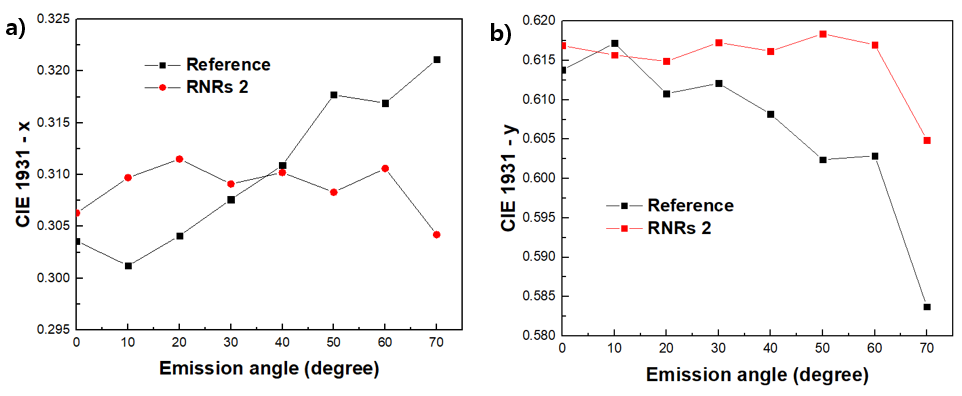


**Supplementary Figure S5**. Viewing-angle-dependent colour coordinates of green phosphorescent OLEDs in CIE 1931 of OLEDs (a) x and (b) y
